# Competitive method-based electrochemiluminescent assay with protein–nucleotide conversion for ratio detection to efficiently monitor the drug resistance of cancer cells†
†Electronic supplementary information (ESI) available: Characterization of the rolling circle amplifications, drug resistance assay with the conventional technologies and supplementary figures and tables. See DOI: 10.1039/c6sc02801b


**DOI:** 10.1039/c6sc02801b

**Published:** 2016-08-04

**Authors:** Wen-Bin Liang, Ming-Zhen Yang, Ying Zhuo, Ying-Ning Zheng, Cheng-Yi Xiong, Ya-Qin Chai, Ruo Yuan

**Affiliations:** a Key Laboratory of Luminescent and Real-Time Analytical Chemistry (Southwest University) , Ministry of Education , College of Chemistry and Chemical Engineering , Southwest University , Chongqing 400715 , PR China . Email: yqchai@swu.edu.cn ; Email: yuanruo@swu.edu.cn; b Department of Clinical Biochemistry , Laboratory Sciences , Southwest Hospital , Third Military Medical University , 30 Gaotanyan Street, Shapingba District , Chongqing 400038 , PR China

## Abstract

A simple and highly-efficient approach to monitor the expression of P-glycoprotein (P-gp) in cells was urgently needed to demonstrate the drug resistance of cancer cells. Herein, a competitive method-based electrochemiluminescent (ECL) assay with a single ECL indicator was proposed for the first time to efficiently estimate the concentration ratio of two proteins. By converting the different proteins to partially coincident nucleotide sequences *via* a sandwich type immunoassay on magnetic beads, the concentration ratio related ECL signals could be obtained *via* competitive nucleotide hybridization on an electrode surface. This method could thoroughly overcome the limitations of simultaneous ECL assays *via* multiple ECL indicators with inevitable cross reactions. At the same time, rolling circle amplification was employed to improve the detection performances, especially the detection limit and sensitivity. With P-gp and glyceraldehyde 3-phosphate dehydrogenase (GAPDH) as a model, the proposed ECL assay was successfully employed to monitor the drug resistance of cancer cells. Compared with conventional technologies, improved sensitivity and accuracy were achieved with a correlation coefficient of 0.9928 and a detection limit of 0.52%. Success in the establishment of the competitive method-based ECL assay offered an efficient strategy to demonstrate the concentration ratio of two proteins and a potential approach for detecting other proteins and nucleotide sequences, revealing a new avenue for ultrasensitive biomolecule diagnostics, especially in cell function research.

## Abstract 2

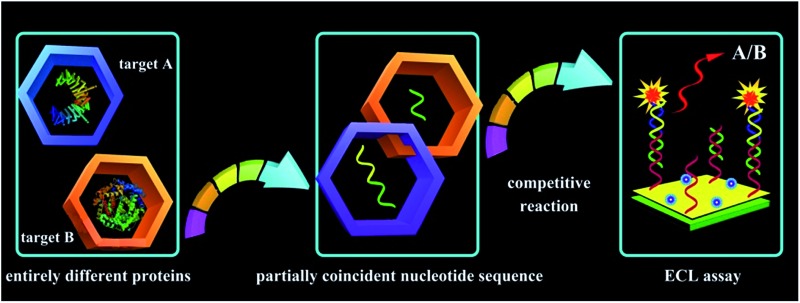
A competitive method-based electrochemiluminescent (ECL) assay with a single ECL indicator was proposed to efficiently estimate the concentration ratio of two proteins.

## Introduction

Currently, using anticancer drugs to regulate cell apoptosis continues to remain at the front line of treatment strategies against cancer.^1^ Unfortunately, a substantial proportion of treatments with anticancer drugs are ineffective due to the drug resistance of the cancer cells *via* various pathways. For instance, the most important and widely present drug resistance mechanism is based on adenosine 5′-triphosphate-dependent drug efflux pumps *via* P-glycoprotein (P-gp).^2^ It is reported that the state of drug resistance positively correlates to the expression of P-gp, and so it has been employed as an “artificial indicator” of drug resistance.^3^ At the early stage of drug resistance with a little increase in the expression of P-gp, the effectiveness of a drug could be recovered by reducing or stopping the medication. However, when the concentration of P-gp was high enough to be detected by the conventional technologies, such as western blotting and confocal laser scanning microscopy, the effectiveness of the drug couldn't be recovered at this stage.^4–7^ Considering the challenge and promising applications, it was urgent to develop an ultrasensitive approach to identify the expression of P-gp in cells and then demonstrate the drug resistance of those cancer cells, especially at that early stage.

As a powerful analytical technique, electrochemiluminescent (ECL) assays have attracted considerable attention due to their low background, wide dynamic range, and cost-effectiveness, and especially for the high sensitivity that is obtained by simple approaches.^8–11^ Various ECL assays have been developed for the sensitive detection of proteins and nucleotide sequences, especially ECL assays with tris(2,2′-bipyridyl)ruthenium(ii) (Ru(bpy)_3_^2+^) and its derivatives as ECL indicators. Chen and co-workers have reported an ECL assay based on tris(2,2′-bipyridyl-4,4′-dicarboxylato)ruthenium(ii) (Ru(dcbpy)_3_^2+^) as the ECL indicator to label nucleotide sequences for ultrasensitive detection of the target nucleotide sequence.^12^ Zhu's group have developed an ECL assay to sensitively determine the activity of the activated cysteine protease *via* streptavidin-modified Ru(bpy)_3_^2+^-doped silica as the signal indicator.^13^ Significant advantages have been obatined for the ultrasensitive detection of proteins, nucleotide sequences and cells, especially when combined with sensitizing approaches employing nucleotide amplification.^14–18^ However the approaches that employ just detection of a single target were not suitable to identify the expression of P-gp in cells accurately due to the difficulty in acquiring proteins from a single cell and calculating the cell numbers. In the available P-gp detection technologies, another stably expressed protein, such as β-actin or glyceraldehyde 3-phosphate dehydrogenase (GAPDH), was essential to be used as a control.^4–7,19–21^ The expression of P-gp was investigated as the concentration ratio between P-gp and the stably expressed protein, such as GAPDH, which could be obtained by monitoring the concentrations of P-gp and GAPDH achieved *via* different ECL assays over multiple runs. Undoubtedly, it was time-consuming and laborious. It was a better choice to obtain these concentrations *via* an ECL assay using different ECL indicators on a single interface in a single run. However, inevitable limitations should be taken into consideration due to the potential cross reactions between these ECL indicators. Very recently, we have proposed a multiparameter analysis-based ECL assay to modestly overcome the limitations from the cross reactions between different ECL indicators.^22^ Although the multiparameter analysis with two ECL indicators was helpful to simultaneously determine the concentration of P-gp and the stably expressed protein, such as GAPDH, to obtain the concentration ratio between these proteins, it was laborious and time-consuming. This was due to the requirement of a massive amount of data to establish the multiparameter model and to eliminate the limitations from the cross reactions between the different ECL indicators. It was a highly valuable assay, but posed further challenges, namely to develop an efficient ECL strategy with a single ECL indicator to achieve the concentration ratio, which would thoroughly overcome the limitations from the cross reactions among multiple ECL indicators. In this regard, an efficient ECL approach with a single ECL indicator was designed based on a competitive method, which was accurate and highly valuable for the early determination of drug resistance of cancer cells *via* investigation of the concentration ratio. To the best of our knowledge, no related research has been reported to estimate the concentration ratio among different proteins based on an ECL assay until now.

Herein, the competitive method-based ECL assay was proposed for the first time to demonstrate the concentration ratio between P-gp and GAPDH by converting these different proteins to partially coincident nucleotide sequences *via* a sandwich type immunoassay on magnetic beads to obtain the concentration ratio related ECL signals *via* competitive nucleotide hybridization on an electrode surface. Specifically, the biotinylated primary antibodies of P-gp and GAPDH were attached onto streptavidin coated magnetic beads (MB@SA), and the nucleotide initiator labeled secondary antibodies were captured in the presence of P-gp and GAPDH by specific antibody–antigen interactions. In order to amplify the responses, the nucleotide initiators were employed to further achieve rolling circle amplification (RCA), which could extend the initiator to a long repeated nucleotide sequence with cascade amplification.^23–26^ And then, these repeated nucleotide sequences from the RCA reactions were enzymatically cut into partially coincident nucleotide sequences, which could competitively react with the capture nucleotide sequences modified on the electrode surface. Just one of these partially coincident nucleotide sequences could capture the signal probes (ECL indicators with labeled nucleotide sequences). Thus, the ECL signals could be employed to demonstrate the competitive reactions and the concentration ratio of these nucleotide sequences and the related target proteins. The success in the establishment of the competitive method-based ECL assay offered an efficient strategy to demonstrate the concentration ratio simply. This method could be readily expanded to electrochemical, fluorescent and chemiluminescent assays for various kinds of proteins and nucleotide sequences, providing an efficient tool for early detection of drug resistance and revealing a new avenue for ultrasensitive biomolecule diagnostics, especially in cell function research.

## Experimental methods

### Preparation of the biotinylated primary antibodies

According to the manufacturer's instructions, the primary antibodies for P-gp and GAPDH were biotinylated based on the reaction between the *N*-hydroxysuccinimide (NHS) group on NHS–biotin and amine groups on the antibodies. Briefly, the appropriate NHS–biotin solution was added into a PBS solution with primary antibodies of P-gp (PAb(P), 1 mg mL^–1^), and the reaction was held at 37 °C for 2 h after complete mixing. Finally, the biotinylated PAb(P) (bio-PAb(P)) was obtained after removing the non-reacted NHS–biotin by careful desalinization. The same protocol was employed to prepare the biotinylated primary antibodies of GAPDH (bio-PAb(G)).

### Preparation of the nucleotide initiator labeled secondary antibodies

The secondary antibodies for P-gp and GAPDH were labeled with a nucleotide initiator based on bioconjugations *via* an EDC/NHS reaction. Briefly, 20 μL of secondary antibodies for P-gp (SAb(P), 1 mg mL^–1^) were firstly mixed with 100 μL of nucleotide initiator for P-gp (NI(P)) by continuous stirring for 10 min. After addition of 100 μL of reaction buffer including 0.20 M 1-ethyl-3-[3-dimethylaminopropyl] carbodiimide hydrochloride (EDC) and 0.05 M sulfo-NHS, the mixture was stirred at room temperature for 2 h to enable full bioconjugation between NI(P) and SAb(P). Further dialysis with centrifugal filter devices (50 kD) was employed to remove the non-reacted NI(P) and related chemicals. The prepared NI(P) labeled SAb(P) was named as SAb(P)-NI(P). Based on the similar protocol as described above, the nucleotide initiator for GAPDH (NI(G)) was labeled onto the secondary antibodies for GAPDH (SAb(G)), and the product was named as SAb(G)-NI(G).

### Preparation of the ECL indicator labeled nucleotide sequences

Ru(dcbpy)_3_^2+^ was employed as the ECL indicator in this work, which was labeled onto the nucleotide sequences as a signal probe based on the EDC/NHS reaction. Briefly, 100 μL of Ru(dcbpy)_3_^2+^ solution (0.10 M) was mixed with 20 μL of nucleotide sequences (50 μM), and then 50 μL of EDC (0.20 M) and 50 μL of sulfo-NHS (0.05 M) was added into the mixture with continuous stirring at room temperature for 2 h for a complete reaction. Immediately, the reacted mixture was purified to remove the non-reacted Ru(dcbpy)_3_^2+^ and related chemicals, and the obtained Ru(dcbpy)_3_^2+^ labeled nucleotide sequences as signal probes (RuSP) were characterized *via* high performance liquid chromatography.

### Cell culture

Human breast cancer MCF-7 cells were purchased from the Type Culture Collection of the Chinese Academy of Sciences (Shanghai, China). MCF-7 cells were grown in Dulbecco's modified Eagle's medium (DMEM) that was supplemented with 10% foetal bovine serum, 1% non-essential amino acids and 10 μg mL^–1^ insulin. The doxorubicin-resistant MCF-7 cells (MCF-7/ADR) with a high expression of P-gp were developed from the parental MCF-7 cells that were subjected to persistent gradient exposure to doxorubicin through increasing doxorubicin concentration from 5 ng mL^–1^ until the cells acquired resistance to 1 mg mL^–1^ of doxorubicin. All of the cells were maintained in a humidified atmosphere with 5% CO_2_ at 37 °C.

As a control, western blotting was performed as followed. Firstly, MCF-7 cells and MCF-7/ADR cells were seeded at different ratios for 24 h, and then the whole-cell lysates were extracted from the cells using a radio-immunoprecipitation assay buffer. Western blotting assays were performed as previously described.^27^ Whole-cell lysates were separated on an 8% SDS-PAGE gel and transferred onto polyvinylidene difluoride (PVDF) membranes. The membranes were blocked for 1 h at 37 °C in 5% non-fat milk/TBST and were then incubated with primary antibodies overnight at 4 °C. The antibodies were used as follows: PAb(P) (1 : 1000) and PAb(G) (1 : 1000). The membrane was then rinsed in TBST and incubated with various secondary antibodies for 2 h at 25 °C. Immunoreactive bands were visualized with a chemiluminescent horseradish peroxidase substrate (Millipore).

Immunofluorescence with a confocal laser scanning microscope was also employed as a control in this study. Briefly, MCF-7 cells and MCF-7/ADR cells were seeded at different ratios for 24 h firstly. After washing with PBS, the cells were fixed in 4% paraformaldehyde for 30 min and blocked in immunol staining blocking buffer for 1 h at 37 °C. Following incubation with SAb(P) (1 : 100), the cells were washed with PBS containing 0.025% Triton X-100 3 times, and then incubated with rabbit anti-goat fluorescein isothiocyanate isomer I (FITC)-conjugated secondary antibodies (1 : 1000) for 1 h at room temperature. The nuclei were stained with 4′,6-diamidino-2-phenylindole (DAPI), and the cells were observed by confocal laser scanning microscopy.

### Measurement procedure

**Scheme 1:**
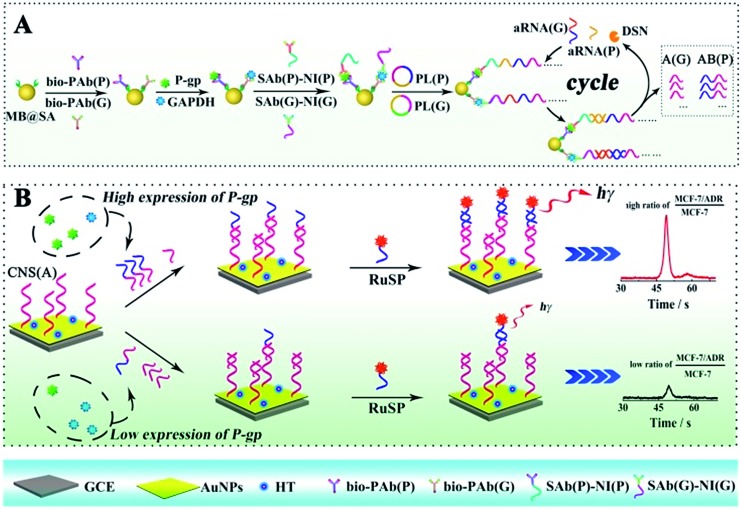
Schematic diagrams of the competitive method-based ECL assay to demonstrate the concentration ratio of P-gp and GAPDH as a model. (A) Schematic diagrams of the sandwich type immunoassay on magnetic beads to convert the different proteins to partially coincident nucleotide sequences; (B) schematic diagrams of the competitive reaction on the sensor surface to obtain the concentration ratio related ECL signals.

The ECL assay was performed based on a sandwiched format immunoassay on magnetic beads and ECL determination on a biosensor as shown in Scheme 1. Before the ECL assay, the biosensor was fabricated as follows. Firstly, the glassy carbon electrode (GCE) was carefully polished with alumina slurries (0.3 and 0.05 μm) and ultrasonicated in deionized water to remove the physically adsorbed materials. After drying with nitrogen gas, the GCE was immersed in 2 mL of HAuCl_4_ solution (10 mg mL^–1^) for electrochemical deposition under a constant potential of –0.2 V for 30 s to obtain a porous gold nanoparticle (AuNP) modified electrode. And then, the AuNP modified GCE was dipped into the solution with the capture nucleotide sequences (CNS(A)) to modify the nucleotide sequences onto the AuNPs. Finally, 1-hexanethiol (HT) solution (0.1 mM) was employed to block the non-specific binding sites on the electrode to obtain the HT/CNS(A)/AuNP modified electrode used for the ECL assay. For the sandwiched format immunoassay on magnetic beads, the biotinylated primary antibodies of P-gp (bio-PAb(P)) and GAPDH (bio-PAb(G)) were attached onto the MB@SA based on the specific interaction between biotin and streptavidin. Then the P-gp, GAPDH and nucleotide sequence labeled detection antibodies of P-gp and GAPDH (SAb(P) and SAb(G), respectively) could be bound onto the magnetic beads *via* specific interactions between the antigens and antibodies by incubating at 37 °C for 40 min. The amount of nucleotide sequence labeled detection antibodies was positively related to the concentration of antigens. After washing twice with PBST to remove the non-reacted secondary antibodies and other related materials, the amplification *via* RCA was performed based on the reaction between the prepared padlocks (PL(P) and PL(G)) in TE buffer with 1 μL of Phi29 polymerase and 5 μL of dNTPs for 6 h at 37 °C. The obtained long nucleotide sequences with repeated sequences were then cleaved into very short nucleotide sequences (AB for P-gp (AB(P)) and A for GAPDH (A(G)), respectively) based on hybridization with assistant nucleotide sequences (aRNA(P) and aRNA(G)) *via* reaction in a Tris–HCl buffer at 95 °C for 5 min and 37 °C for 40 min, and specific cleavage with DSN *via* addition of 10 μL of DSN enzyme at 65 °C for 15 min and 50 μL of DSN stop solution at 65 °C for 5 min. The obtained nucleotide sequences (AB(P) and A(G)) were dropped onto the fabricated electrode surface and the reactions were maintained at 37 °C for 40 min for the competitive hybridization of AB(P) and A(G) to capture the nucleotide sequences (CNS(A)) modified on the electrode surface. The resultant biosensor was incubated with signal probes (RuSP) at 37 °C for 40 min. After every reaction, the electrode was carefully washed with Tris–HCl buffer. Finally, the ECL responses of these biosensors were investigated in PBS with 25 mM tripropylamine (TPrA).

## Results and discussion

### Electrochemical characterization of the biosensor

**Fig. 1:**
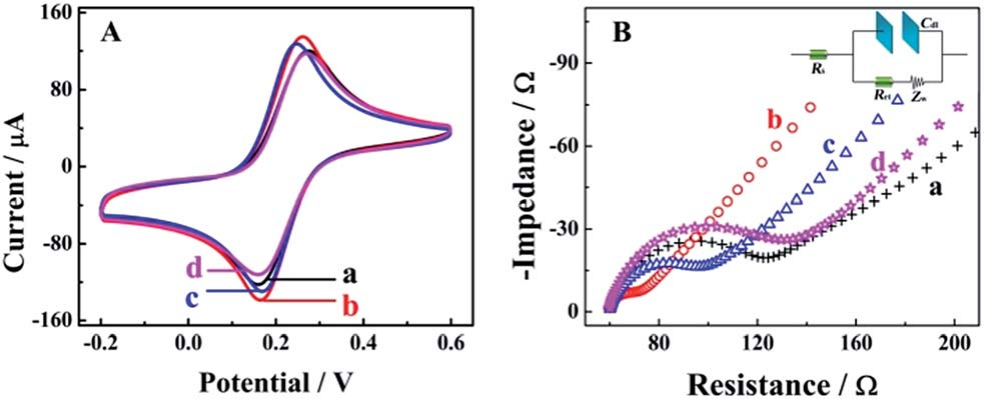
Characterization of biosensors with different fabrications based on CV (A) and EIS (B); curve a, bare GCE; curve b, AuNPs/GCE; curve c, CNS(A)/AuNPs/GCE; curve d, HT/CNS(A)/AuNPs/GCE. Inset, the equivalent circuit with model impedance data of EIS responses.

Cyclic voltammetry (CV) and electrochemical impedance spectroscopy (EIS) measurements were employed as useful electrochemical technologies to characterize the fabrication of the biosensor step by step (Fig. 1). As shown in the Fig. 1A for CV measurements in 0.1 M PBS with 5 mM [Fe(CN)_6_]^3–/4–^ at the potential range from 0.2 to 0.6 V, there was a pair of well-defined redox peaks for [Fe(CN)_6_]^3–/4–^ for the bare GCE (curve a), indicating good electron transfer on the electrode surface. When the AuNPs were electrodeposited onto the electrode surface, a higher peak current and smaller oxidation–reduction potential were observed due to the good electron transfer ability and conductivity of the AuNPs (curve b, AuNPs/GCE). An obviously decreased peak current could be observed after the modification of capture nucleotide sequences onto the AuNPs (curve c, CNS(A)/AuNPs/GCE), because the nonconductive nucleotide sequences would decrease the electron transfer. For the similar property of HT, the peak current was decreased furthermore after blocking with HT (curve d, HT/CNS(A)/AuNPs/GCE). Further characterization *via* EIS in 0.1 M PBS with 5 mM [Fe(CN)_6_]^3–/4–^ at a frequency range from 5 × 10^–2^ to 1 × 10^6^ Hz is shown in Fig. 1B in the form of a Nyquist plot. In the EIS measurements, the electron transfer resistance (*R*_et_) was employed to demonstrate the electron transfer kinetics quantitatively, which was similar to the semicircle diameter in the EIS response in the form of a Nyquist plot and was calculated accurately based on the equivalent circuit with model impedance data (inset of Fig. 1B). As shown in curve a for the bare GCE, a normal EIS response was obtained with *R*_et_ of 53.2 Ω. After electrodeposition of the AuNPs onto the electrode surface (curve b, AuNPs/GCE), the *R*_et_ was decreased obviously to 19.6 Ω, indicating the good electron transfer on the electrode surface. When the capture nucleotide sequences were modified onto the AuNP surface (curve c, CNS(A)/AuNPs/GCE) and blocked with HT (curve d, HT/CNS(A)/AuNPs/GCE), the *R*_et_ was increased to 68.3 Ω and 96.7 Ω, respectively. These EIS results indicated the same tendency of electron transfer as that obtained by CV. All the results of these EIS and CV measurements confirmed the successful fabrication of the proposed biosensor.

### Mechanism of competitive method-based ECL assay

For the competitive detection of the ratio between P-gp and GAPDH, it is necessary to construct a competitive relationship between these entirely different proteins. Considering the favorable designability and various available amplification approaches of nucleotide sequences, the secondary antibodies of P-gp and GAPDH (SAb(P) and SAb(G)) were firstly labeled with two special nucleotide sequences respectively. Based on a sandwiched format immunoassay on magnetic beads, the amount of secondary antibodies was increased with the increasing concentrations of P-gp and GAPDH respectively, which could be demonstrated as *c*_NI(P)_ ∝ *c*_P_ and *c*_NI(G)_ ∝ *c*_G_, where *c*_NI(P)_ and *c*_NI(G)_ are the concentrations of the nucleotide labels on the secondary antibodies of P-gp and GAPDH respectively, and *c*_P_ and *c*_G_ are the concentrations of P-gp and GAPDH respectively. Due to amplification *via* RCA reactions and specific cleavage *via* DSN, the nucleotide labels on the detection antibodies of P-gp and GAPDH could be amplified significantly to give two similar nucleotide sequences (named as A(G) and AB(P) respectively), where A(G) and AB(P) contained some of the same sequences as shown in Table S1† and the nucleotide sequence on AB(P) and A(G) could be employed for hybridization with the capture nucleotide sequence (named as CNS(A)) on the electrode surface. Thus, the concentrations of A(G) and AB(P) were positively related with the concentrations of the detection antibodies of GAPDH and P-gp, respectively, which could be demonstrated as *c*_AB(P)_ ∝ *c*_NI(P)_ ∝ *c*_P_ and *c*_A(G)_ ∝ *c*_NI(G)_ ∝ *c*_G_, where *c*_AB(P)_ and *c*_A(G)_ are the concentrations of the amplified nucleotide sequences AB(P) and A(G), respectively. The obtained AB(P) and A(G) could be competitively reacted with CNS(A) on the electrode surface and section B on AB(P) could capture the signal probe (RuSP) to generate the ECL signal. Thus, the reactions could be demonstrated using the following kinetic equations:

1 CNS(A) + A(G) = CNS(A)A(G)

2 CNS(A) + AB(P) = CNS(A)AB(P)

3
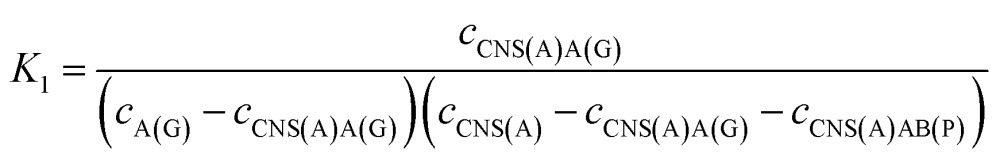

4
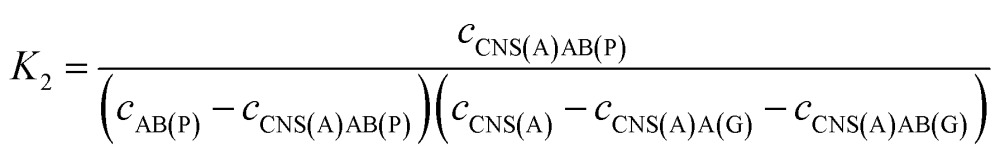



When the concentration of CNS(A) was much lower than that of A(G) and AB(P), the obtained AB(P) and A(G) would competitively react with CNS(A). Thus, the concentrations of CNS(A)A(G) (*c*_CNS(A)A(G)_) and CNS(A)AB(P) (*c*_CNS(A)AB(P)_) would be directly related to the ratio of *c*_AB(P)_ and *c*_A(G)_ and be illustrated in the following equations calculated from eqn (3) and (4):5
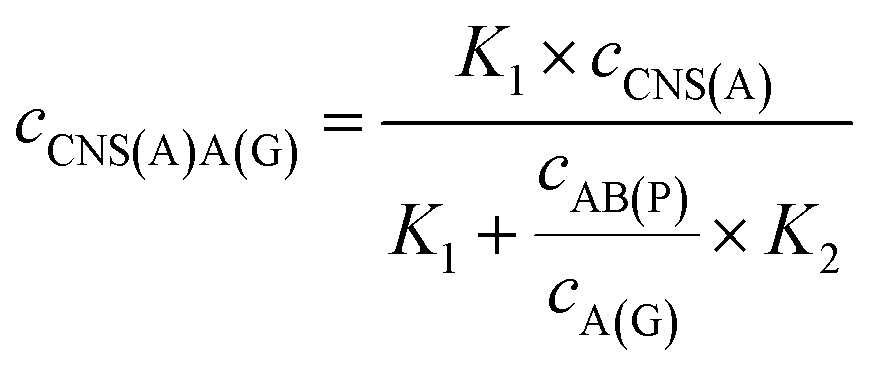

6
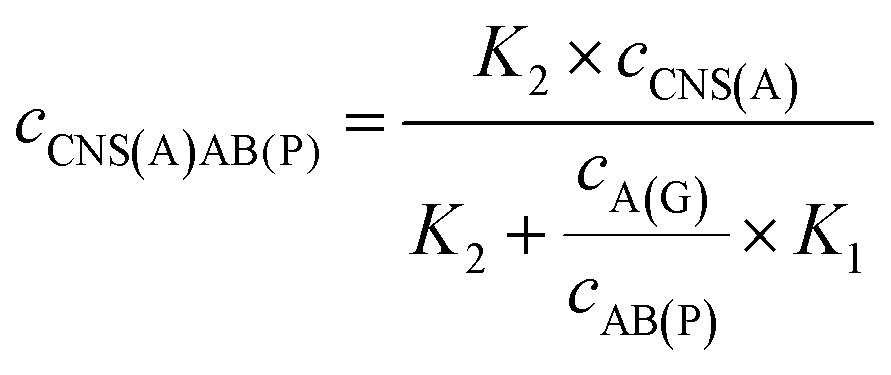
where the *c*_CNS(A)_, *K*_1_ and *K*_2_ are all fixed values in the system. Due to the ECL intensity (Int_ECL_) being positively related to *c*_CNS(A)AB(P)_ and there being an unavoidable background ECL signal (Int_BG_), the equation could be modified into the following equations:7
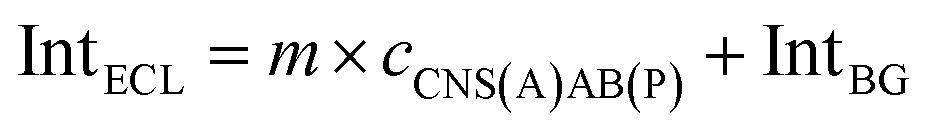

8
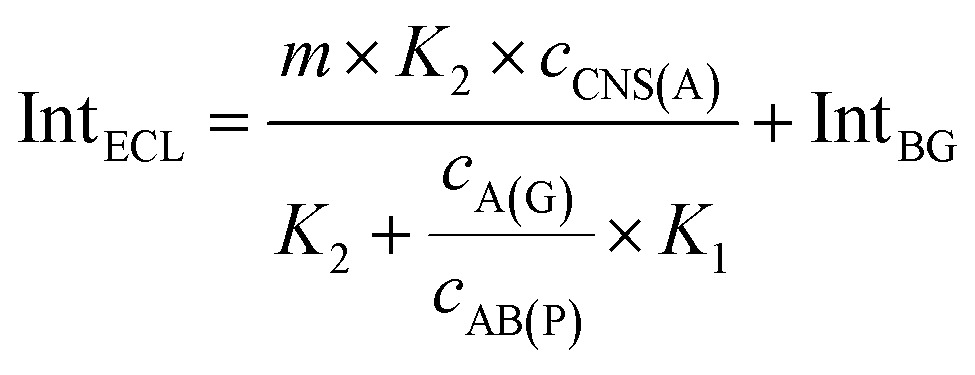
where *m* is the coefficient between the ECL intensity and *c*_CNS(A)AB(P)_. Thus, the ratio of the two entirely different proteins, P-gp and GAPDH, could be detected and calculated simply based on this mechanism and the proposed model. Furthermore, this approach was also suitable to investigate the ratio of other analytes, such as proteins, nucleotide sequences and cells.

### Electrochemical and ECL responses of the biosensor

**Fig. 2:**
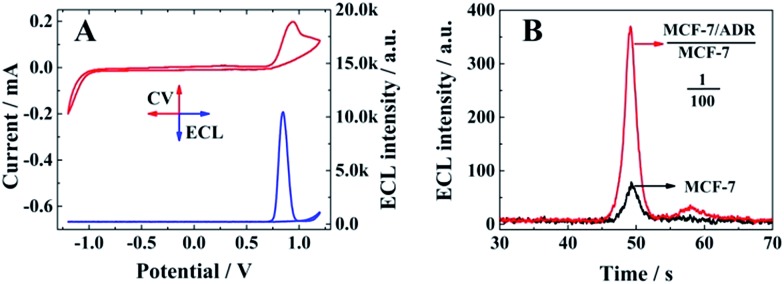
(A) CV (red line) and ECL–potential (blue line) results of the biosensor in PBS with 25 mM TPrA as coreactant (MCF-7/ADR : MCF-7 = 1 : 2); (B) ECL–time signals of the competitive method-based ECL assay for MCF-7 cells (black line) and MCF-7 cells with MCF-7/ADR cells (MCF-7/ADR : MCF-7 = 1 : 100) (red line).

The electrochemical (red line) and ECL responses (blue line) of the biosensor are shown in Fig. 2A, when the ratio between MCF-7/ADR and MCF-7 was 1 : 2. A significant ECL emission with the peak current at 0.85 V could be obtained with the oxidation of Ru(dcbpy)_3_^2+^ and TPrA, indicating the presence of a signal probe (RuSP) on the biosensor surface due to the specific nucleotide hybridization. Furthermore, MCF-7 cells and MCF-7 cells with MCF-7/ADR cells were employed to confirm the relationship between the ECL emission and presence of MCF-7/ADR based on the competitive method-based ECL assay. As shown in Fig. 2B, there was a maximum ECL emission at about 370 a.u. for the MCF-7 cells with MCF-7/ADR cells (MCF-7/ADR : MCF-7 = 1 : 100), which was significantly higher than that of MCF-7 cells without MCF-7/ADR cells, indicating that the proposed competitive method-based ECL assay could be employed to demonstrate the presence of cancer cells with drug resistance based on the detection of the concentration ratio between P-gp and GAPDH.

### Application of the proposed competitive method-based ECL assay

**Fig. 3:**
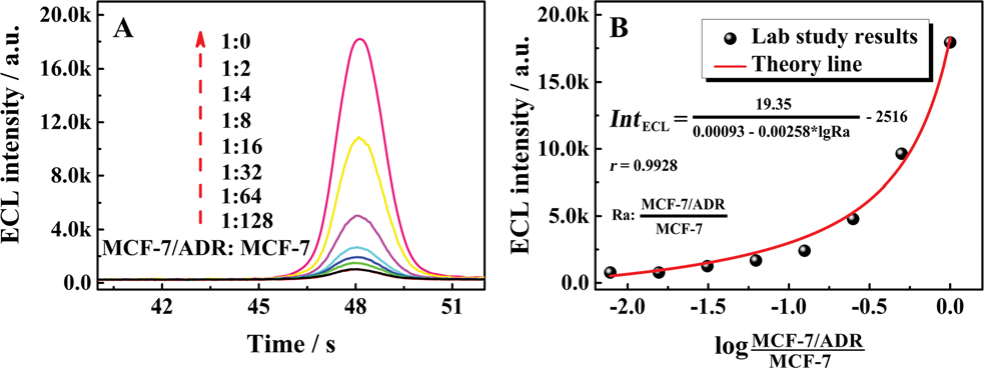
(A) ECL–time responses of the as-proposed competitive method-based ECL assay for different ratios of MCF-7/ADR with MCF-7; (B) calibration curve for the relationship between the ECL intensity and the logarithm of the ratio of MCF-7/ADR : MCF-7.

To estimate the performance of the as-proposed competitive method-based ECL assay for quantitative identification of the drug resistance of cancer cells, the ratio between P-gp and GAPDH in MCF-7 cells with a different ratio of MCF-7/ADR (MCF-7/ADR : MCF-7) was detected in the range from 1 : 128 to 1 : 0. As shown in Fig. 3A with the ECL–time responses of the proposed ECL assay, the ECL emissions were increased with the ratio of MCF-7/ADR : MCF-7. The relationship between the ECL emissions and the ratio of MCF-7/ADR : MCF-7 could be fitted well with the demonstrated theory line according to the competitive strategy. The fitted theory line could be calculated as 
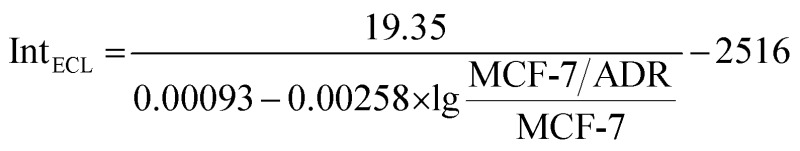
 with a correlation coefficient of 0.9928 and a detection limit of 0.52%, where Int_ECL_ is the ECL intensity obtained by the competitive method-based ECL assay (Fig. 3B) and the detection limit was down to 52 MCF-7/ADR cells with drug resistance in 1 × 10^4^ normal MCF-7 cells. At the same time, a limit of quantification of 0.86% was obtained, indicative of a good performance for quantitative determination with suitable precision and accuracy. Therefore, the as-proposed strategy could be employed to demonstrate the ratio of MCF-7/ADR : MCF-7 based on the ratio of P-gp to GAPDH obtained *via* the competitive method-based ECL assay, indicating the acceptable application of the as-proposed competitive method-based ECL assay to monitor the drug resistance of cancer cells quantitatively.

Additionally, as important properties for the ECL assay, the reproducibility, repeatability and stability of the assay were evaluated for duplicate measurements in this work. No more than 5% for the relative standard deviation (RSD) was obtained for all of the reproducibility, repeatability and long time stability studies, specifically 4.8%, 3.2% and 4.6% respectively, indicating the satisfactory application performance of the competitive method-based ECL assay.

**Fig. 4:**
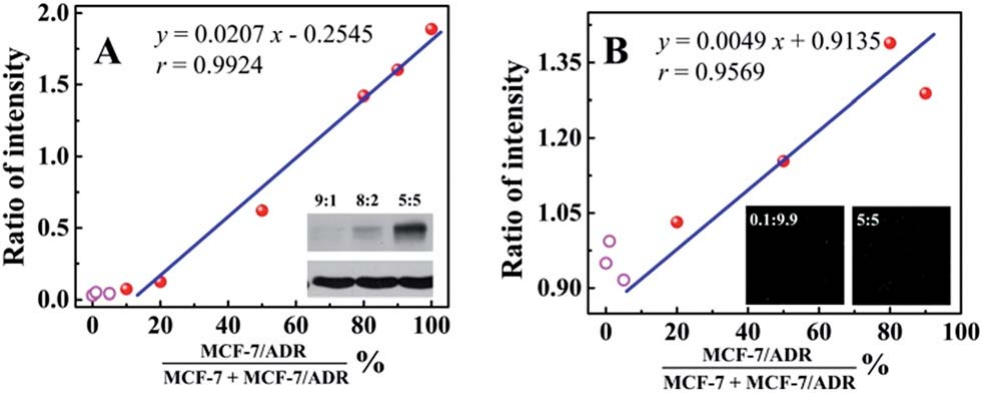
(A) Calibration curve for the relationship between the western blotting responses and the ratio of MCF-7/ADR with MCF-7 (insert, the image of the western blotting results of MCF-7/ADR with MCF-7 with ratios of 9 : 1, 8 : 2, and 5 : 5, respectively); (B) calibration curve for the relationship between the immunofluorescence results with confocal laser scanning microscopy and the ratio of MCF-7/ADR with MCF-7 (insert, the image obtained by confocal laser scanning microscopy of MCF-7/ADR with MCF-7 with ratios of 9.9 : 0.1, and 5 : 5, respectively).

In order to evaluate the applicability of the competitive method-based ECL assay compared with the conventional technologies used to monitor the drug resistance of cancer cells, western blotting and immunofluorescence with confocal laser scanning microscopy were employed to detect MCF-7 cells with different ratios of MCF-7/ADR cells (Fig. 4). Detailed information and results of these studies are shown in the ESI.† As shown in Fig. 4A with the calibration curve for the relationship between the western blotting responses and the ratio of cancer cells with drug resistance, MCF-7/ADR, the ratio of intensity obtained from the gray level analysis was increased with the increasing ratio of cancer cells with drug resistance, with a correlation coefficient of 0.9924 and a detection limit of 13.99%. For the immunofluorescence results using confocal laser scanning microscopy (Fig. 4B), a better detection limit of 6.07% could be obtained. Whereas, a lower correlation coefficient (*r* = 0.9569) was obtained due to the narrow response range and low sensitivity. Compared with the conventional technologies used to identify the drug resistance of cancer cells, improved sensitivity and accuracy were achieved based on the as-proposed competitive method-based ECL assay, especially the correlation coefficient of 0.9928 and detection limit of 0.52%. This is indicative of the good performance and the potential application of the as-proposed ECL assay to monitor cancer cells with drug resistance and demonstrate their levels quantitatively.

## Conclusions

In summary, a competitive method-based ECL assay was developed to demonstrate the concentration ratio of entirely different proteins and identify the drug resistance of cancer cells. The strategy was processed by a sandwich type immunoassay on magnetic beads to convert the proteins to partially coincident nucleotide sequences. Competitive nucleotide hybridization on the electrode surface would then result in an ECL signal related to the concentration ratio of the target proteins and the drug resistance of the cancer cells. With P-gp and GAPDH as a model, satisfactory performances were achieved to identify the drug resistance of the cancer cells based on the proposed ECL assay, which was fitted well with the theory line with a correlation coefficient of 0.9928 and a detection limit of 0.52%. The improved sensitivity and accuracy compared with the conventional technologies indicated the good performance and potential application of the as-proposed ECL assay to identify the drug resistance of cancer cells and demonstrate their levels quantitatively. Most importantly, the competitive method-based ECL assay offered an efficient strategy to demonstrate the concentration ratio simply, which could be readily expanded for various kinds of proteins and nucleotide sequences. This efficient strategy also provided an efficient tool for the early detection of drug resistance and revealed a new avenue for ultrasensitive biomolecule diagnostics, especially in cell function research.

## Abbreviation list

AB(P): Product AB for P-gp
A(G): Product A for GAPDH
aRNA(G): Assistant RNA for GAPDH
aRNA(P): Assistant RNA for P-gp
AuNPs: Gold nanoparticles
bio-PAb(G): Biotinylated primary antibodies for GAPDH
bio-PAb(P): Biotinylated primary antibodies for P-gp
CNS(A): Capture nucleotide sequences
CV: Cyclic voltammetry
CV%: Variation coefficients
DAPI: 4′,6-Diamidino-2-phenylindole
DMEM: Dulbecco's modified Eagle's medium
dNTPs: Deoxynucleotides
DSN: Duplex-specific nuclease
EDC: 1-Ethyl-3-[3-dimethylaminopropyl] carbodiimide hydrochloride
ECL: Electrochemiluminescent
EIS: Electrochemical impedance spectroscopy
FITC: Fluorescein isothiocyanate isomer I
GAPDH: Glyceraldehyde 3-phosphate dehydrogenase
GCE: Glassy carbon electrode
HT: 1-Hexanethiol
HPLC: High performance liquid chromatography
MB@SA: Streptavidin coated magnetic beads
MCF-7/ADR: Doxorubicin-resistant MCF-7 cells
NHS–biotin: Biotin *N*-hydroxysuccinimide ester
PBS: Phosphate buffer saline
PBST: PBS buffer with Tween 20
P-gp: P-Glycoprotein
PL(P): Padlock for P-gp
PL(G): Padlock for GAPDH
PVDF: Polyvinylidene difluoride
RCA: Rolling circle amplification
RSD: Relative standard deviations
RuSP: Ru(dcbpy)_3_^2+^ labeled signal probe
Ru(bpy)_3_^2+^: Tris(2,2′-bipyridyl)ruthenium(ii)
Ru(dcbpy)_3_^2+^: Tris(2,2′-bipyridyl-4,4′-dicarboxylato)ruthenium(ii)
SAb(G)-NI(G): Nucleotide initiator labeled secondary antibodies for GAPDH
SAb(P)-NI(P): Nucleotide initiator labeled secondary antibodies for P-gp
sulfo-NHS: *N*-Hydroxysulfosuccinimide
TBST: Tris-buffered saline with Tween 20
TE: Tris-buffered saline with EDTA
