# Supplementary material for: Competitive method-based electrochemiluminescent assay with protein–nucleotide conversion for ratio detection to efficiently monitor the drug resistance of cancer cells
Source: Chem Sci. 2016 Aug 4;7(12):7094–100. doi: 10.1039/c6sc02801b (PMC5355943; doi:10.1039/c6sc02801b)
Supplement: Supplementary file 1 [file SC-007-C6SC02801B-s001.pdf]

Electronic Supplementary Material (ESI) for Chemical Science.
This journal is © The Royal Society of Chemistry 2016

Competitive Method-Based Electrochemiluminescent Assay with Protein-nucleotide Conversion for Ratio Detection to Efficiently Monitor Drug Resistance of Cancer Cells

Wen-Bin Liang,[†, ‡] Ming-Zhen Yang,[‡] Ying Zhuo,[†] Ying-Ning Zheng,[†] Cheng-Yi Xiong,[†] Ya-Qin Chai,[*, †] and Ruo Yuan[*, †]

[†] Key Laboratory of Luminescent and Real-Time Analytical Chemistry (Southwest University), Ministry of Education, College of Chemistry and Chemical Engineering, Southwest University, Chongqing 400715, PR China

[‡] Department of Clinical Biochemistry, Laboratory Sciences, Southwest Hospital, Third Military Medical University, 30 Gaotanyan Street, Shapingba District, Chongqing 400038, China

Table of Contents

Reagents and materials. S3

Table S1. ....S 4

Apparatus. ....S 5

Optimization of the AuNPs deposition. ....S 5

Figure S1 ....S 6

ECL mechanism of the proposed ECL assay. ....S 7

Characterization of the Rolling Circle Amplifications. ....S 7

Figure S2 ....S 8

Drug Resistance Assay with the Conventional Strategies. ....S 8

Figure S3 ....S 9

Figure S4 ....S 10

Reagents and materials.

$Ru(dcbpy)_3^{2+}$ was purchased from Suna Technology Inc. (Suzhou, China). N-hydroxysulfosuccinimide (sulfo-NHS), 1-ethyl-3-[3-dimethylaminopropyl] carbodiimide hydrochloride (EDC), $HAuCl_4$, biotin N-hydroxysuccinimide ester (NHS-biotin), tripropylamine (TPrA), streptavidin coated magnetic beads (MB@SA), Triton X-100, 1-hexanethiol (HT), fluorescein isothiocyanate isomer I (FITC) and 4',6-diamidino-2-phenylindole (DAPI) were obtained from Sigma Chemical Co. (MO, USA). Dulbecco's modified eagle's medium (DMEM), fetal bovine serum, penicillin and streptomycin were purchased from Gibco Laboratories Life Technologies Inc. (Grand Island, NY, USA). The primary antibodies and secondary antibodies for P-gp and GAPDH were obtained from Proteintech Group (Chicago, IL, USA). Rabbit anti-goat FITC-conjugated secondary antibodies were purchased from ZSGB-Bio Inc. (Beijing, China). T4 ligase, Phi29 polymerase and duplex-specific nuclease (DSN) were purchased from Vazyme Biotech Co., Ltd. (Nanjing, China). Polyvinylidene difluoride (PVDF) membranes were purchased from Millipore Co. (Darmstadt, Germany). Deoxynucleotides (dNTPs) was obtained from Genview Scientific Inc. (EI Monte, CA, USA). Exconuclease I and Exconuclease III were purchased from Thermo Fisher Scientific Inc. (Shanghai, China). Radio-immunoprecipitation assay buffer, tris-buffered saline with EDTA (TE) buffer, tris-buffered saline with tween 20 (TBST), phosphate buffer saline (PBS) buffer, PBS buffer with tween 20 (PBST), Immunol Staining Blocking Buffer and paraformaldehyde were obtained from Beyotime Institute of Biotechnology (Jiangshu, China). Ultrapure water with a resistivity of 18.2 MΩ/cm was used throughout this study.

All oligonucleotides were custom-synthesized by Shanghai Sangon Biological Engineering Technology and Services Co., Ltd. (Shanghai, China). The sequences information was listed as Table 1. Before using, the circular templates were mixed with the primers, heated to 65 °C for 10 min, and then reacted with T4 ligase after cooling to room temperature to link 5' and 3' end of these circular templates. The non-reacted circular templates and primers were cleaned by adding 5 μL Exconuclease I and 2 μL Exconuclease III and heating to 37 °C for 30 min. The Exconuclease I and Exconuclease III were deactivated on 90 °C for 15 min.

Table S1. The oligonucleotides list employed in the competitive method-based ECL assay.

Name	Sequence (5'→3')
nucleotide initiator for GAPDH / NI(G)	PO_4-TCA GAG TCT TTC CTA CTC CTA CTG TTT TT-NH_2
padlock for GAPDH / PL(G)	PO_4-GAA AGA CTC TGA TGT CCT CGA GAT GAT GCA GTA GGA GTA G
assistant RNA for GAPDH / aRNA(G)	CAG UAG GAG UAG GAA AGA CUC UGA
product A for GAPDH / A(G)	CTA CTC CTA CTG CAT CAT CTC GAG GAC ATC AGA GTC TTT C
nucleotide initiator for P-gp / NI(P)	PO_4-TCA GAG TCT TTC ACA GGA CTC CTA TTT TT-NH_2
padlock for P-gp / PL(P)	PO_4-GAA AGA CTC TGA TGT CCT CGA GAT GAT GCA GTA GGA GTA GGC ATC GTG ATC CGA TAG GAG TCC TGT
assistant RNA for P-gp / aRNA(P)	UAG GAG UCC UGU GAA AGA CUC UGA
product AB for P-gp / AB(P)	*ACA GGA CTC CTA TCG GAT CAC GAT GC*C TAC TCC TAC TGC ATC ATC TCG AGG ACA TCA GAG TCT TTC
Ru(dcbpy)$_3^{2+}$ labeled signal probe / RuSP	*GCA TCG TGA TCC GAT AGG AGT CCT GT*T TTT T-NH_2
capture nucleotide sequences / CNS(A)	NH_2-TTT TTT TTT TTT TTT GA*T GTC CTC GAG ATG ATG CAG TAG GAG TAG*

(The difference between NI(G) and NI(P) was shown with blue and red color respectively. The difference between AB(P) and A(G) was shown with underlined italic letters. The double underlined letters of CNS(A) referred to the sequences hybridized with AB(P) and A(G). The wavy underlined letters of RuSP referfed to the sequences just hybridized with AB(P).

Apparatus.

Electrochemistry and ECL measurements were carried out with a model MPI-E II multifunctional electrochemical and chemiluminescent analytical system (Xi'an Remax Electronic Science and Technology Co., Xi'an, China) with a conventional three-electrode system, where a Ag/AgCl was employed as the reference electrode, a platinum wire was employed as auxiliary electrode, and a glassy carbon electrode (GCE) with/without modification was employed as the working electrode, respectively. The purification and characterizations of the ECL materials labeled nucleotide sequences were performed on ultimate 3000 high performance liquid chromatography system (HPLC, Thermo Scientific, PA, USA). Laser confocal microscopy measurements were performed with FITC labelled P-gp antibodies and DEPI by laser confocal microscope (Leica Microsystem Inc., Heidelbery, Germany).

Optimization of the AuNPs deposition.

For the electrodeposition of AuNPs, the deposition time was a main factor, which was optimized with different electrodeposition time and characterized by SEM. As shown in the Figure S1, the particle size and amount of the particles were increased with the increasing electrodeposition time from 10 s to 60 s. When the deposition time was larger than 30 s, there were some agglomerated particles, which may decrease the special characters of nano-materials.

Thus, the 30 s was employed as the optimized electrodeposition time for the modification of AuNPs on the electrode surface.

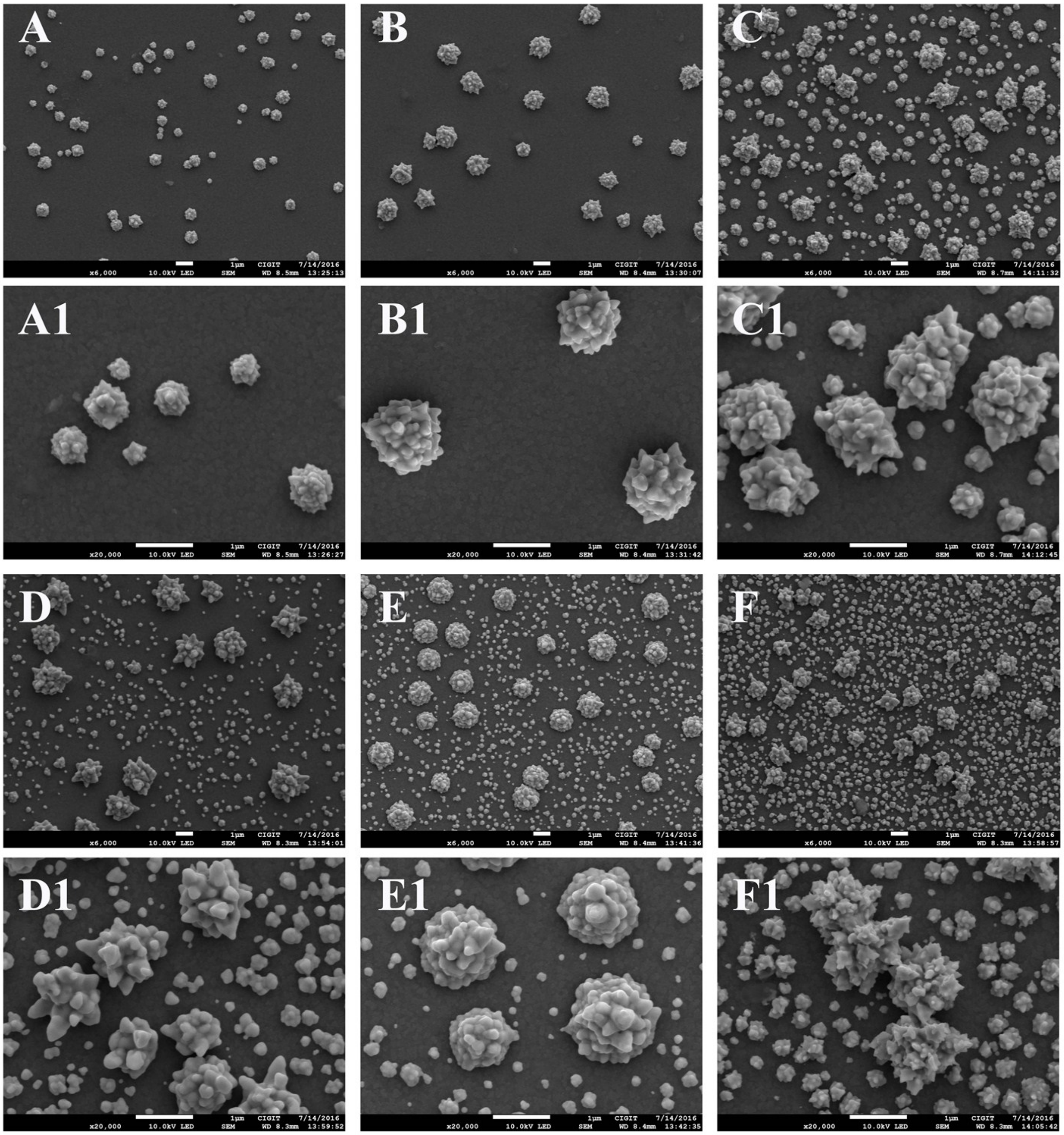

Figure S1 Optimization of the AuNPs deposition time. A, 10 s; B, 20 s; C, 30 s; D, 40 s, E, 50 s; F, 60s; A1-F1 were the local enlarged figures of A-F.

ECL mechanism of the proposed ECL assay.

Many different co-reactants could enhance the ECL emission of $Ru(bpy)_3^{2+}$ system with different mechanism, in which amine was the most widely used co-reactant. For the $Ru(bpy)_3^{2+}$ system with amine, such as TPrA as co-reactant used in the as-proposed ECL assay, the mechanism was formulated as Scheme S1, which included three main reactions: (1) the oxidation of secondary amine (R−NH−R) of co-reactant to radical cation ($R{-}N^{\bullet+}H{-}R$) and $Ru(dcbpy)_3^{2+}$ to $Ru(dcbpy)_3^{3+}$; (2) the generation of the excited form of $Ru(dcbpy)_3^{*2+}$ by the reaction between radical cation ($R{-}N^{\bullet+}H{-}R$) and $Ru\text{-}(dcbpy)_3^{3+}$; (3) ECL emission when $Ru(dcbpy)_3^{*2+}$ went back to $Ru(dcbpy)_3^{2+}$.

$$Ru(bpy)_3^{2+} - e \longrightarrow Ru(bpy)_3^{3+} \quad (1)$$
$$R_2NCH_2R - e \longrightarrow R_2N^{+\bullet}CH_2R \quad (2)$$
$$Ru(bpy)_3^{3+} + R_2NCH_2R \longrightarrow Ru(bpy)_3^{2+} + R_2N^{+}\dot{C}H_2R \quad (3)$$
$$R_2N^{+\bullet}CH_2R \longrightarrow R_2N^{\bullet}CH_2R + H^{+} \quad (4)$$
$$Ru(bpy)_3^{3+} + R_2N^{\bullet}CH_2R \longrightarrow [Ru(bpy)_3^{2+}]^{*} + R_2N^{+}{=}CHR \quad (5)$$
$$[Ru(bpy)_3^{2+}]^{*} \longrightarrow Ru(bpy)_3^{2+} + \text{light} \quad (6)$$

Scheme S1 Schematic illustration for the mechanism model of the proposed $Ru(bpy)_3^{2+}$-based ECL assay.

Characterization of the Rolling Circle Amplifications.

Polyacrylamide gel electrophoresis could provide a versatile and high resolution method to characterize the nucleotides according to their molecular weight and electrophoretic mobility, which was employed to characterize the efficiency of the amplification approach in this work, specially rolling circle amplification (RCA) and specific enzymatic splicing *via* DSN. As shown in Figure S2, the molecular weight of the studied nucleotides could be investigated by the distant places of UV band from the notch. The nucleotides after RCA reactions from initiators (lane 1 and 2 respectively) showed high molecular weights (> 2000 bp) due to the long nucleotide

sequence with massive repeated sequences. After specific enzymatic splicing *via* DSN, the long nucleotide sequence could be spliced to large amount of special small nucleotide sequences (lane 3 and 4 respectively), indicating the successful transformation from single initiator to massive special nucleotide sequences with high efficiency, which was important and useful to amplify the response for the ultrasensitive assay.

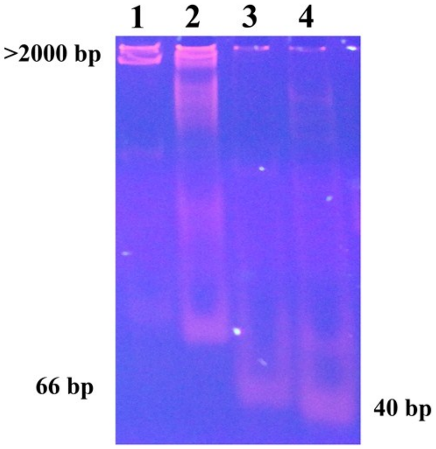

Figure S2. Polyacrylamide gel electrophoresis characterization of the rolling circle amplifications and efficiency of the enzymatic splicing *via* DSN.

Drug Resistance Assay with the Conventional Strategies.

To validate the performance of the proposed competitive method-based ECL assay for early detection of drug resistance, herein, the expression of P-gp was detected by the conventional strategies, specially western blotting with glyceraldehyde 3-phosphate dehydrogenase (GAPDH) as the control protein and confocal laser scanning microscope. As shown in the western blotting image of P-gp and GAPDH expression (Figure S3), the expression of GAPDH was on a stable level, whereas the expression of P-gp was increased with the ratio of MCF-7/ADR with MCF-7. Based on the gray level analysis, the expression of P-gp and GAPDH could be evaluated based on the gray values, and then the concentration ratio between P-gp and GAPDH could be

calculated quantitatively as shown in Figure 4A with correlation coefficient of 0.9924 and detection limit of 13.99%.

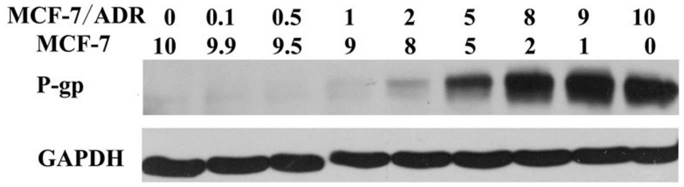

Figure S3. Western blotting image for the proteins expression (P-gp and GAPDH, respectively) for different ratio of MCF-7/ADR: MCF-7 cells.

For the confocal laser scanning microscope images of the various ratio of MCF-7/ADR with MCF-7, blue and green color could be observed from DAPI stained cell nuclear and fluorescein isothiocyanate isomer I (FITC) labeled P-gp antibodies, respectively (Figure S4). In addition, these images were overlaid as the merged confocal images (MERGE) to confirm the location of these fluorescent responses. The fluorescent responses from FITC labeled P-gp antibodies were increased with the ratio of MCF-7/ADR: MCF-7 obviously. To quantize the expression level of P-gp *via* these fluorescent responses, an Fluorescent Index was calculated with ten different images by equation 1, in which the signal degree was the fluorescent intensity of FITC labeled P-gp antibodies with 0 - 4 levels.[1, 2]

$$\text{Fluorescent Index} = \sum \frac{Cell\ Number_i \times Signal\ degree}{Totle\ Cell\ Number} \quad \text{eq. 1}$$

As shown in Figure 4B, the Fluorescent Index increased with the ratio of MCF-7/ADR with MCF-7 with correlation coefficient of 0.9569 and detection limit of 6.07%.

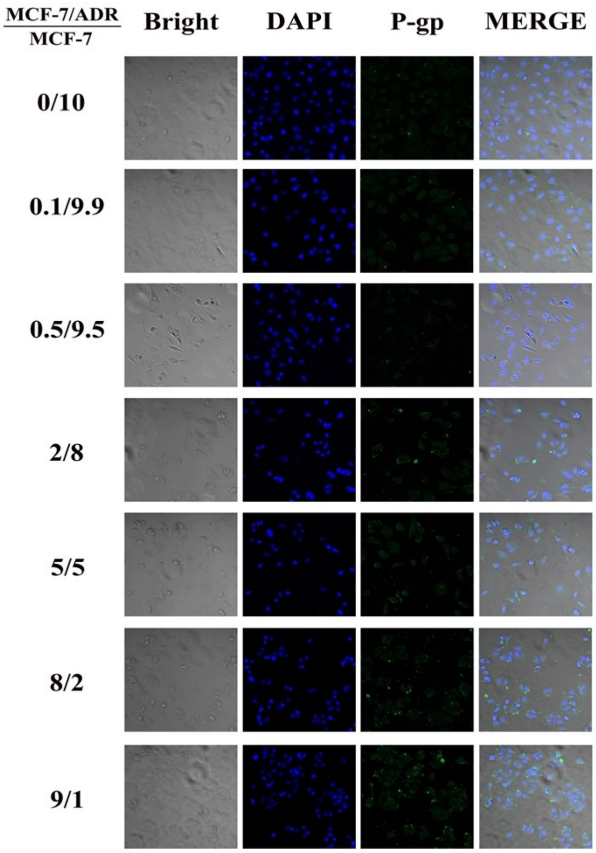

Figure S4. Laser confocal microscope image for various ratio of MCF-7/ADR with MCF-7 cells with different proteins expression of P-gp.

REFERENCES

(1) Zhang, Z. J.; Wang, Q. L.; Li, P.; Zhou, Y.; Li, S. H.; Yi, W. J.; Chen, A.; Kong, P. Y.; Hu, C. M. *Cancer Epidemiol.* **2009**, 2, 130-136.

(2) Liang, W. B.; Fan, C. C.; Zhuo, Y.; Zheng, Y. N.; Xiong, C. Y.; Chai, Y. Q.; Yuan, R. *Anal. Chem.* **2016**, 88, 4940-4948.
